# Intratumor heterogeneity index of breast carcinomas based on DNA methylation profiles

**DOI:** 10.1186/s12885-019-5550-3

**Published:** 2019-04-05

**Authors:** Emanuel M. Campoy, María T. Branham, Luis S. Mayorga, María Roqué

**Affiliations:** 1 IHEM-CONICET, Av del libertador, 80 Mendoza, Argentina; 2 Facultad de Ciencias Médicas, Av del Libertador 80, Universidad Nacional de Cuyo, Mendoza, Argentina; 3 Facultad de Ciencias Exactas y Naturales, Padre Jorge Contreras 1300, Universidad Nacional de Cuyo, Mendoza, Argentina

**Keywords:** Intratumor heterogeneity - promoter methylation, TCGA - heterogeneity index - breast Cancer - cellular clones, Prognosis and predictive factors

## Abstract

**Background:**

Cancer cells evolve and constitute heterogeneous populations that fluctuate in space and time and are subjected to selection generating intratumor heterogeneity. This phenomenon is determined by the acquisition of genetic/epigenetic alterations and their selection over time which has clinical implications on drug resistance.

**Methods:**

DNA extracted from different tumor cell populations (breast carcinomas, cancer cell lines and cellular clones) were analyzed by MS-MLPA. Methylation profiles were used to generate a heterogeneity index to quantify the magnitude of epigenetic heterogeneity in these populations. Cellular clones were obtained from single cells derived of MDA-MB 231 cancer cell lines applying serial limiting dilution method and morphology was analyzed by optical microscopy and flow cytometry. Clones characteristics were examined through cellular proliferation, migration capacity and apoptosis. Heterogeneity index was also calculated from beta values derived from methylation profiles of TCGA tumors.

**Results:**

The study of methylation profiles of 23 fresh breast carcinomas revealed heterogeneous allele populations in these tumor pieces. With the purpose to measure the magnitude of epigenetic heterogeneity, we developed an heterogeneity index based on methylation information and observed that all tumors present their own heterogeneity level. Applying the index calculation in pure cancer cell populations such as cancer cell lines (MDA-MB 231, MCF-7, T47D, HeLa and K-562), we also observed epigenetic heterogeneity. In addition, we detected that clones obtained from the MDA-MB 231 cancer cell line generated their own new heterogeneity over time. Using TCGA tumors, we determined that the heterogeneity index correlated with prognostic and predictive factors like tumor size (*p* = 0.0088), number of affected axillary nodes (*p* = 0.007), estrogen receptor expression (*p* < 0.0001) and HER2 positivity (*p* = 0.0007). When we analyzed molecular subtypes we found that they presented different heterogeneity levels. Interestingly, we also observed that all mentioned tumor cell populations shared a similar Heterogeneity index (HI) mean.

**Conclusions:**

Our results show that each tumor presents a unique epigenetic heterogeneity level, which is associated with prognostic and predictive factors. We also observe that breast tumor subtypes differ in terms of epigenetic heterogeneity, which could serve as a new contribution to understand the different prognosis of these groups.

## Background

It has been estimated that 14.1 million new cancer cases and 8.2 million cancer deaths occurred in 2012 worldwide [1]. Breast cancer ranks as the most frequent cancer in women in less developed regions, contributing with 25% of the new cases diagnosed in 2012 [1]. Normally, breast cancers of advanced stages have poorer prognosis [2].

Cancer is a disease defined by alterations at the genomic, epigenomic, transcriptomic and proteomic level. The interplay between these events triggers the acquisition of cancer hallmarks which, by occurring in different cells, evolve and can constitute a heterogeneous tumor cell population. The populations fluctuate in space and time and are subjected to selection [3]. It is reasonable therefore to sustain that intratumor heterogeneity (ITH) is determined by two events: first, by the acquisition of genetic/epigenetic alterations; and secondly, through their selection over time [4]. Although the ITH is frequently related to the spatial organization of cells in a tumor, it is also necessary to consider the temporal dimension for the evolution of cancer cell populations. One of the models that synthetizes spatial and temporal heterogeneity of tumors is “the clonal evolution model of cancer” proposed by Peter Nowell in 1976 [5]. This model proposed that endogenous and exogenous factors induce mutational processes providing the fuel for genetic variation between cancer cells, which determine selection of cancer cell populations [3]. This evolutionary model was useful to understand tumor growth and treatment failure and also contributed to reveal the increased tumor aggressiveness that occurs during the natural history of advanced solid tumors [6].

Most of the current therapies treat cancer as a homogenous disease, which has clinical implications on drug resistance. It has been described that anti-neoplasic drugs act specifically on cellular clones with, for example, mutated oncogenes, leaving other populations lacking these mutations unaltered. In this way the untargeted clones, can proliferate and maintain tumor progression after the drug treatment [7] [8]. It has also been determined that continued drug exposure produces selection of the surviving cells, which increases the evolution rate and allows induction of more aggressive clones with new properties [9]. So far there is enough evidence to consider the study of ITH a key feature to enhance treatment strategies.

During cancer progression, aberrant promoter methylation of tumor suppressor genes (TSG) is one of the most common alterations in solid tumors [10] and has been described as an early event in many tumor types, including breast tumors [11] [12] [13]. The CpG sites located in these TSG are normally unmethylated in healthy cells, but frequently methylated in cancer cells. In fact, several tumor types (or tumor subtypes) present an specific methylation profile [14] [15]. Methylation has a direct impact on different pathways of cancer hallmarks, including DNA repair, cell cycle regulation and evading apoptosis [16] [17]. Although epigenetic modifications are dynamic, some marks remain through cancer progression [18] [19]. Considering this, the epigenetic information configurates the epigenomic landscape of each tumor.

Distinct methods have been developed to study genetic ITH: deep-sequencing, multi-region sequencing and single cell DNA sequencing [20] [21] [22] [23]. Several studies also consider the genomic information to infer ITH which is afterward associated with prognostic or predictive factors [24]. For example, breast cancer patients with low genomic ITH were more likely to have complete pathological response to neoadjuvant chemotherapy [25]. In this work we propose to quantify ITH using a mathematical approach to calculate a heterogeneity index (HI) from methylation profiles of breast tumors, cancer cells lines and public datasets.

## Methods

### Patients and tumor samples

Twenty three patients treated in the Gineco-Mamario Institute of Mendoza with breast cancer were enrolled after obtaining their informed consent based on the scientific and ethical principles of the World Medical Association’s Declaration of Helsinki. Ethical approval was obtained from the Ethics Committee of the Faculty of Medical Sciences, from the National University of Cuyo, Mendoza, Argentina. All tumors presented clinical-pathological data, i.e. tumor type, tumor stage (T, N, M), side, tumor grade, mitotic index, patient age, ER expression, PR expression, HER2 expression). A database containing the clinical-pathological information and DNA methylation profiles was generated.

### Materials

Dulbecco’s Modified Eagle Medium (D-MEM), RPMI Medium and Penicillin-Streptomycin were obtained from Gibco Laboratories (Thermo Fisher Scientific, Argentina) and fetal bovine serum was obtained from Internegocios S.A. Mercedes, Buenos Aires. DNA extraction kits (PureLink® Genomic DNA, Thermo Fisher Scientific). MS-MLPA kits manufactured by MRC-Holland.

### Cell culture

MDA-MB 231, MCF7, T47D and HeLa human cells lines were grown in D-MEM while K562 cell line was grown in RPMI. All cell lines were supplemented with 10% fetal bovine serum, penicillin/streptomicin at 37 °C in an atmosphere of 95% air and 5% CO2, in T-25 flasks and 12, 24 and 96 plates to 80% confluence.

### Cell cloning by serial dilution

MDA-MB 231 cells were used, the initial population was trypsinized and counted using a Neubauer chamber to prepare a cell suspension of 1000 cells/ml. Serial dilutions were generated to obtain 1 cell in 200 μl of complete D-MEM media and this volume was added per well in a 96 plate in order to obtain a unique cell for each well. Individual cells grew up to 80% of confluence.

### DNA extraction

Fresh tissues from breast invasive ductal carcinomas (IDC) and surgical margins were frozen at − 80 °C and fragmented with a frozen mortar. The homogenate was collected and suspended in PBS buffer. All samples were stored for at least 24 h at − 20 °C and DNA was collected as previously described (tumor pieces and surgical margins) [26]. Cancer Cell lines and cellular clones were trypsinized and centrifuged (except K-562 that grow in suspension). After homogenization and centrifugation (cellular pellets), DNA extraction was carried out with specific kit (PureLink® Genomic DNA, Thermo Fisher Scientific).

### Methylation assays by methyl specific- multiplex ligation probe amplification (MS-MLPA)

The methylation status of 98 CpG sites located in 54 genes related to cancer was tested in DNA obtained from: IDCs, surgical margins, cancer cell lines (MDA-MB 231, MCF-7, T47D, K-562 and HeLa) and clones derived from MDA-MB 231 by MS-MLPA. Commercial kits ME001, ME002, ME003 and ME011 were used (MRC Holland, Amsterdam, The Netherlands). Reactions were carried out following manufacturer’s instructions, including subtle modifications to avoid background signals [15]. Fluorescent PCR products were separated by capillary electrophoresis in a Beckman CEQ8000 sequencer (Beckman Coulter Inc. Fullerton, CA, USA) and analyzed with GeneMarker v1.75 software (Softgenetics LLC, PA, USA).

### Heterogeneity index calculation

**Fig. 1:**
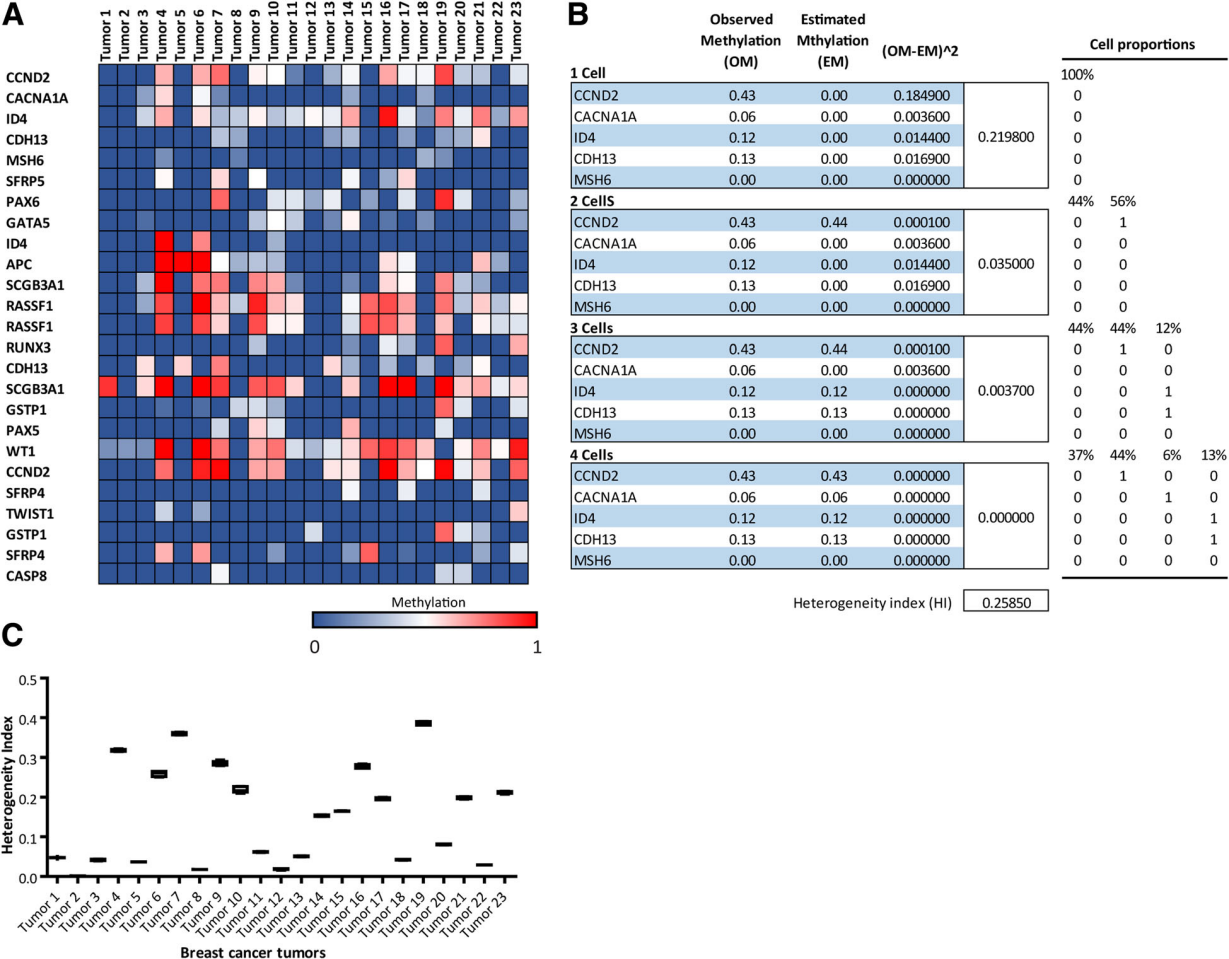
Heterogeneity index of breast carcinomas inferred from methylation profile obtained by MS-MLPA**. a.** Heat map showing the methylation status of 25 CpG sites (19 TSG) in rows on 23 breast tumors, represented in columns. A color gradient from blue-white-red is used to represent low to high values of methylation (from 0 to 1). **b.** Scheme showing the heterogeneity Index calculation using 1, 2, 3 and 4 cells inferred from methylation status of 5 CpG sites of tumor 7 (OM) showed in A. All the possible populations and all the possible cell proportions were tested in order to fit the observed values. The match between experimental and estimated methylation values (EM) was only seldom perfect. Considering the experimental error, we defined the sub-HI as the sum of the squared deviation between the experimental and estimated values (OM-EM)^2. The tumor final HI was set to the average of sub-HI after five randomized selections. **c.** HI calculated for the 23 breast tumors represented in box plot graph. The HIs are statistically different as assed by ANOVA with Tukey post hoc test *p* < 0.0001

To calculate the HI for the methylation observed in five CpG sites, we combined theoretical populations having pure methylated [1] and non-methylated (0) values in different proportions to reproduce the experimental values. The questions were, how many alleles, which alleles, and in what proportions are necessary to explain the epigenetic heterogeneity observed in a cellular population? The criterion to select the most suitable populations and the best proportions of these populations was to minimize the sum of the squared difference between the adjusted and the observed values for the five sites (for an example, see Fig. 1B and the corresponding explanation in the Results section). This value was calculated using from 1 to 4 populations. The final HI was the sum of these 4 values. The rationale for this criterion was that the HI was very small when the observed values could be adjusted with just a single population. In contrast, the HI was high when the adjusted and the observed values were very different, and more and more populations were needed to find a combination that reproduced the observed values. To select the best populations and the best proportions of each population to fit the observed value, all the possible populations (2^5 = 32) and all the possible proportions were tested. Proportion is a continuous variable; hence, to make the calculations reasonably fast, the proportion of each population to be tested were limited to 1/16 steps (6.25%). For example, for a single population it is just 100% of one of the 32 possible populations that best fitted the data. For two population, all the possible combinations of 2 of the 32 populations were tested in proportions going from 0 to 1 in 1/16 steps (combinations tested: 0–100%, 6.25–93.75%, 12.5–87.5%, …, 87.5–12.5%, 93.75–6.25%, 100–0%). The use of a limited set of proportions introduces some limitation to the adjustment to the observed values. The heterogeneity index calculation is available at: https://heterogeneidad.herokuapp.com/

### Migration assays

MDA-MB 231 clones were cultured in 12 well plates. Cells were incubated overnight in serum-reduced medium containing 0.5% FBS. Cell surface was then scratched using a sterile 200 μL pipet tip and washed with PBS to remove detached cells and debris. Cells were photographed every 12 h using a TE300 Eclipse microscope equipment (Nikon, Tokyo, Japan). Images were then processed using Image J software. Reduction of the scratched area was measured and expressed as migration percentage according to the formula: ((Areat0-Areat1)/Areat0) × 100.

### Cellular proliferation assay

MDA-MB 231 clones were plated in 96 well plate and MTT assay was performed. After 24 h or 48 h of proliferation, medium containing 1 mg/mL MTT was added to the cells (MDA-MB231 clones) up to a final concentration of 0.5 mg/mL and incubated at 37 °C for 4 h. The medium was then aspired, and the formazan product was solubilized with DMSO. The absorbance at 630 nm (background absorbance) was subtracted from measures at 570 nm for each well (3 replicates for each dilution were performed).

### Flow cytometry

MDA-MB231 clones were seeded in 6 well plates, 37 °C and 5% CO_2_. After 24 h cells were harvested by trypsinization and washed with PBS. After centrifugation, the cells were suspended in 200 μl of PBS and analyzed by flow cytometry (Model BD FACSAria III). Forward and side scatter measurement was registered in order to detect information about the internal cellular complexity (i.e. granularity) and cell size.

### Apoptosis assay

MDA-MB231 clones were seeded in 6 well plates, 37 °C and 5% CO_2_. After 24 h cells were exposed to UV radiation for 30 min and were harvested by trypsinization and washed with PBS. After centrifugation, the cells were suspended in 100 μl of binding buffer and then stained with FITC-annexin V and propidium iodide. After they reacted under dark for 10 min, the cells were resuspended in 100 μl binding buffer and analyzed by flow cytometry (Model BD FACSAria III).

### Database analyses to study the association between HI and clinical-pathological features

DNA methylation status of IDCs was retrieved from the public dataset TCGA Breast Cancer (BRCA) using the UCSC Xena browser (https://xenabrowser.net). HI calculation was performed applying the developed mathematical model considering 15 CpG sites. Association between HI and clinic-pathologic traits was analyzed in 250 TCGA tumors.

### Statistical analysis

ANOVA analysis was applied to detect significant differences in migration, proliferation, apoptosis and HI calculation assays between distinct tumor, cellular and MDA-MB 231 clone populations. Tukey post-hoc test was applied to find out differences in the mean of population groups. GraphPad Prims v5.3 was used for ANOVA calculation of HI calculation. For association studies between clinical-pathological variables and fresh tumors or TCGA tumors, correlation analyses were performed using the software SPSS v17 (SPSS Inc., Chicago, IL, USA) and the software GraphPad Prims V5.3.

## Results

### Methylation heterogeneity for 25 CpG sites in invasive ductal carcinomas

We established a cohort of 23 fresh tumor fractions derived from patients with invasive ductal breast cancer (Table 1). The tumor piece for analysis was selected by optical microscopy by a specialized anatomopathologist, excluding non-tumoral tissue as much as the technique allowed. After DNA isolation the methylation profile of 98 CpG sites located within 54 cancer related genes was determined by Methyl Specific Multiplex Ligation Probe Amplification assay (MS-MLPA). This is a semi-quantitative method, which calculates for each CpG site the proportion of methylated copies normalized to the total amount of copies of each site in the sample. The 54 analyzed genes are distributed across all the autosomal chromosomes and none are methylated in leukocytes, as assessed in our previous studies [26]. With the purpose of define the HI from the most frequently methylated sites, we chose 25 which were methylated in more than 10% of the samples.

**Table 1:** Patient and Tumor characteristics

		Number
Total Patients		23
Total tumors for methylation analyses		23
Age (years)*	≤40	5
	> 40	14
	Unknown	4
Tumors with complete anatomo-pathological data		22
Axillar Lymph Node Status	Positive	9
	Negative	14
Tumor Grade	I	1
	II	5
	III	17
Disease Stage	1	10
	2A	4
	2B	4
	3A	3
	3B	0
	3C	1
	Unknown	1
Molecular Subtypes	Luminal A	3
	Luminal B	16
	HER2	2
	TN	2

*Mean age: 51.31 years (SEM = 1.70)

HER2: Human Epidermal Growth Factor Receptor 2; TN: Triple Negative

For these 25 CpG sites (included in Table 2), all the 23 tumors presented a unique methylation signature (Fig. 1A). Interestingly, the proportion of methylated alleles presented intermediate values between 0 and 1 which indicated that the cellular population was not epigenetically homogeneous. The values could result from a mixture of alleles derived from different cell populations from the tumor, and/or from other cells like immune cells, cancer associated fibroblasts, etc. (tumor microenvironment). For example, the methylation for CpG site CCND2 in Tumor 7 was 0.43, which means that 43% of the CCND2 alleles in the tumor fraction were methylated and 57% were not (see Fig. 1B). Notice that this value cannot be obtained from a pure clonal population, where the only possible values would be 0.5 (for heterozygous genomes), 0 or 1 (for homozygous genomes). So, we can therefore say that a methylation proportion of 0.43 means that CCND2 alleles are heterogeneously methylated in the sample of Tumor 7, which implies that the cellular population is a mixture composed by different tumor cells or tumor cells and stromal cells in which some CCND2 alleles are methylated and others are not. Conversely, the methylation proportion of CCND2 in the sample of Tumor 8 was 0 and this value is compatible with a homogeneous allele population.

**Table 2:** 60 CpG sites analyzed by MS-MLPA

CpG sites respect to ATG	Gene Name (HGDB)	Chromosome Location	CpG sites respect to ATG	Gene Name (HGDB)	Chromosome Location
72 bp to exon 2	APC	5q22	346 bp before	MGMT	10q26.3
4658 bp before	ATM	11q23	93 bp before	MGMT	10q26.3
157 bp after	CACNA1A	19p13.2	151 bp after	MGMT	10q26.3
32 bp before	CACNA1G	17q21.33	382 bp before	MGMT	10q26.3
8560 bp before	CASP8	2q33.2	74 bp after ex 1	MGMT	10q26.3
1168 bp before	CCND2	12p13.3	464 bp after	MGMT	10q26.3
1358 bp before	CCND2	12p13.3	661 bp before	PAX5	9p13
17 bp before	CD44	11p12	49 bp before	PAX6	11p13
411 bp before	CD44	11p12	43 bp before	PRDM2	1p36.21
42 bp before	CDH13	16q23.3	190 nt after	PYCARD	16p11.2
20 bp before	CDH13	16q23.3	651 bp before	RARB	3p24.2
31 bp after	CDKN2A	9p21.3	824 bp before	RARB	3p24.2
407 nt before	CHFR	12q24	888 bp before	RARB	3p24.2
400 nt before	CHFR	12q24	141 bp before	RASSF1	3p21.3
714 bp before	DAPK1	9q22	79 bp before	RASSF1	3p21.3
366,437 bp after	DLC1	8p22	18 bp before	RUNX3	1p36.11
366,993 bp after	DLC1	8p22	232 bp before	SCGB3A1	5q35
163 bp after	ESR1	6q25.1	17 bp before	SCGB3A1	5q35
112 bp after	ESR1	6q25.1	42 bp before	SFRP4	7p14.1
658 bp before	GATA5	20q13.3	82 bp after	SRFP4	10q24.1
103 bp after	GSTP1	11q13	275 bp before	SFRP5	7p14.1
245 bp before	GSTP1	11q13	316 bp before	SFRP5	10q24.1
11 bp before ex 1	HIC1	17p13	172 bp before	TIMP3	22q12.3
163 bp after	HTLF	3q25.1	300 bp before	TIMP3	22q12.13
953 bp before	ID4	6p22.3	10,905 bp before	TP53	17p13.1
319 bp before	ID4	6p22.3	29,790 bp before	TP73	1p36.32
305 bp before	IGSF4	11q23	29,551 bp before	TP73	1p36.3
72 bp before	IGSF4	11q23	220 bp after	TWIST1	7p21.2
464 nt after	KCNQ1	11p15.5	80 nt before	VHL	3P25
432 bp before	MGMT	10q26.3	412 bp before	WT1	11p13

Summarizing, methylation proportions different from 0 and 1 (except 0.5 value that may be due to a heterozygous genomes), indicate heterogeneous allele populations in the tumor piece. Hence, the methylation profile of the 25 analyzed CpG sites had some information about the epigenetic ITH.

### Generation of ITH index based on methylation profiles

With the purpose to measure the magnitude of epigenetic heterogeneity in a sample, we aimed to develop a heterogeneity index (HI) based on the methylation profiles obtained by MS-MLPA. We decided to use 5 CpG sites, a number that proved to be suitable to measure heterogeneity keeping the calculation relatively simple. The basis for the development of the index was to find the smallest number of populations having pure methylated [1] and non-methylated (0) combinations that could explain the observed methylation profile in the sample. For example, if the MS-MLPA of five CpG sites renders the following methylation numbers: 0, 0, 0, 0, 0.32, these values can be explained by the combination of two populations: 68% of 0, 0, 0, 0, 0 plus 32% of 0, 0, 0, 0, 1. If, however, the methylation numbers are: 1, 0.8, 0.4, 0.2, 0, two population are not enough, and three populations are required: 20% of 1, 0, 0, 1, 0 plus 40% of 1, 1, 0, 0, 0 plus 40% of 1, 1, 1, 0, 0. When this criterion was used with real methylation values obtained by MS-MLPA, we found that the match between experimental and estimated values was only seldom perfect. Taking into consideration the experimental error and some limitation in the estimated values (see, M&M), we decided to define HI as the sum of the squared deviation between the experimental and estimated values when using 1 to 4 populations (Fig. 1B). For simplicity’s sake, we are considering cell populations having the two allelic CpG sites methylated or not methylated. Hence, the HI is pooling together intra and inter cellular methylation heterogeneity.

### Invasive ductal breast carcinomas present different heterogeneity index

We aimed to apply the developed HI to compare heterogeneity among tumors basing the analysis on the same CpG sites. For this, we used five clusters of five randomly selected CpG sites (25 CpGs). The average of these cluster values was considered as sub-HI. Since the conformation of the five clusters was randomly selected, we wondered if that could influence the index. Hence, we repeated the analysis several times changing the randomized selections. We observed that the dispersion among different sub-HI of a tumor was relatively small (coefficient of variation in the 0–9.72% range); therefore, the sub-HI average converged when the number of iterations was about 5. Consequently, the tumor’s final HI was set to the average of five sub-HI. When this index was calculated, striking differences were observed among the tumors (ANOVA *p* < 0.0001). The average and SD HI value of the tumor cohort was 0.15 ± 0.12 (Fig. 1C). It is clear that tumors with more methylated sites had higher HI. However, notice that there were differences that were not easy to observe, for example the methylation patterns of tumor 4 and 6 looked similar, but their HI were significantly different. The broad range of HI observed in this cohort of IDC analyzed suggests that it can be considered a tumor feature.

### Cancer cell lines show methylation heterogeneity

We were aware that the methylation profile obtained from fresh tumor pieces could contain epigenetic information derived from microenvironment cells. We ignored, however, the precise stromal proportion in the fresh tumor fractions and therefore its potential impact on the methylation estimations. In order to evaluate methylation heterogeneity in pure cancer cell populations, we added an in-vitro model and analyzed the methylation profile of different breast cancer cell lines (i.e. MDA-MB 231, MCF-7 and T47D), cervical (HeLa) and leukemia (K-542).

**Fig. 2:**
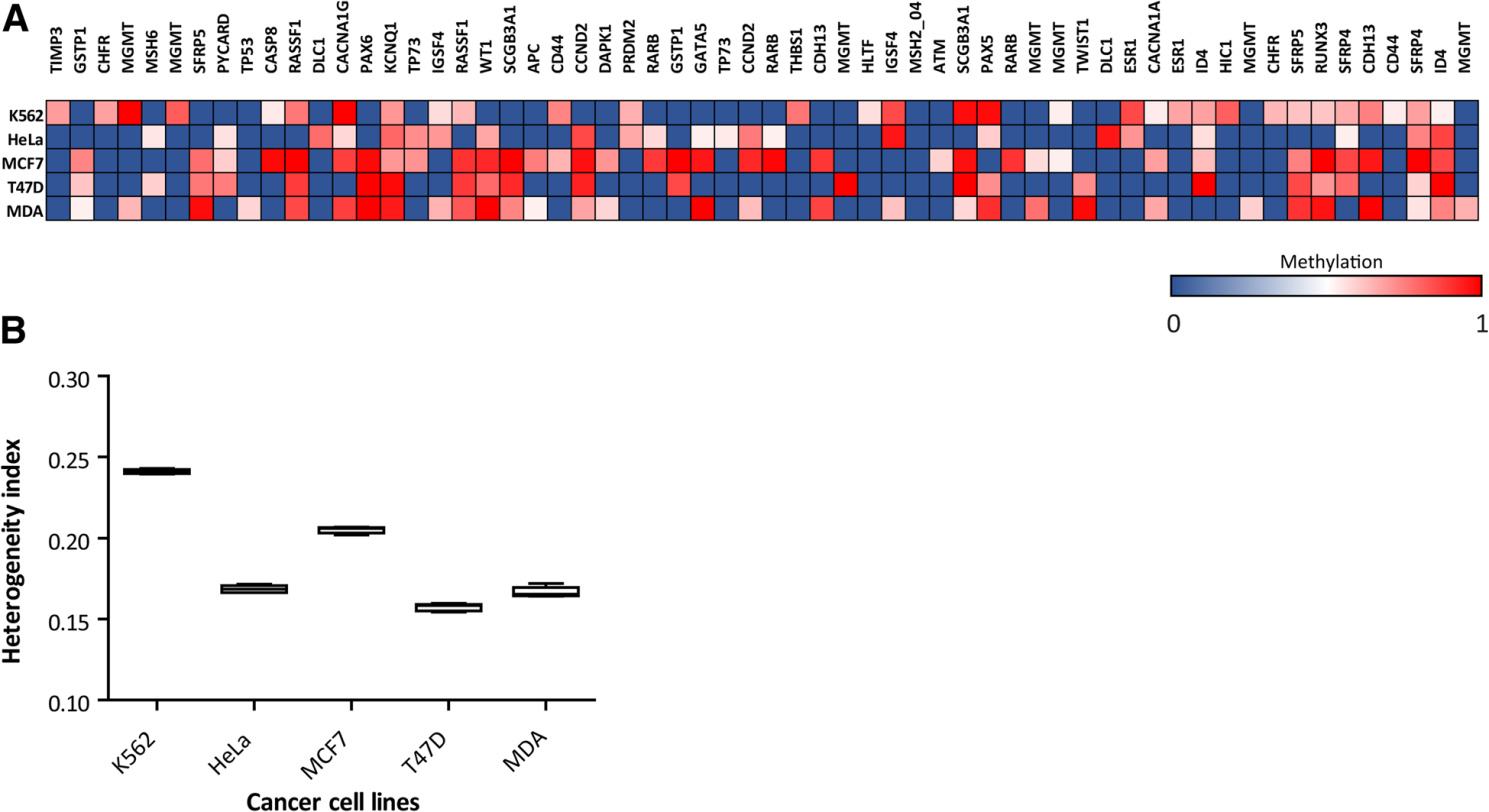
Heterogeneity index of cancer cell lines inferred from methylation profile obtained by MS-MLPA. **a.** Heat map showing the methylation status of 60 CpG sites (in columns) on 5 cancer cell lines (K-562, HeLa, MCF7, T47D and MDA-MB 231), represented in rows. A color gradient from blue-white-red is used to represent low to high values of methylation (from 0 to 1). **b.** HI calculated for the 5 cancer cell lines represented in box plot graph. The HIs are statistically different as assed by ANOVA with Tukey post hoc test *p* < 0.0001

Interestingly, most of the analyzed CpG sites showed intermediate methylation values, which was indicating again the presence of certain heterogeneity within the cell lines (Fig. 2A). Given that these samples lack of stromal derived alleles, these results revealed that the cancer cell lines are composed by different sub-clonal populations which are heterogeneous in epigenetic terms. It is important to note that, just like the 23 analyzed tumors, each cancer cell line presented a different and specific methylation profile.

Next, we calculated the HI values. In this opportunity, the number of methylated CpG sites in more than 10% of the samples ascended to 60 sites. We defined 12 random clusters of 5 CpG sites (60 CpG sites) and calculated the HI of each cell line. Using ANOVA with post-hoc Tukey analysis we observed significant differences (*p* < 0.0001) in the HI among the cell lines (Fig. 2B). K-562 cells (leukemia) presented the highest heterogeneity with an HI = 0.24. The HI calculated for the other cell lines was, HeLa = 0.16, MCF7 = 0.20, T47D = 0.15 and MDA-MB231 = 0.16. The average and SD HI value of the tumor cohort was 0.18 ± 0.03.

These results show that stable cancer cell lines present epigenetic heterogeneity, suggesting that the cell populations are composed by diverse cellular clones. It is worth mentioning that part of the heterogeneity observed may be due to aneuploidy, which is frequent in cancer cell lines.

### Cellular clones derived from MDA-MB 231 present diverse morphology and behavior traits

Considering the epigenetic heterogeneity observed in different populations derived from cancer cell lines, we decided to focus on MDA-MB 231 since this cell line showed an intermediate heterogeneity index (HI = 0.16). We asked whether this cell population could be separated in cellular clones that could explain the observed heterogeneity. For this, using a simple protocol of cell cloning by serial limiting dilution, an initial population of MDA-MB 231 (IP) was diluted to plate individual cells.

**Fig. 3:**
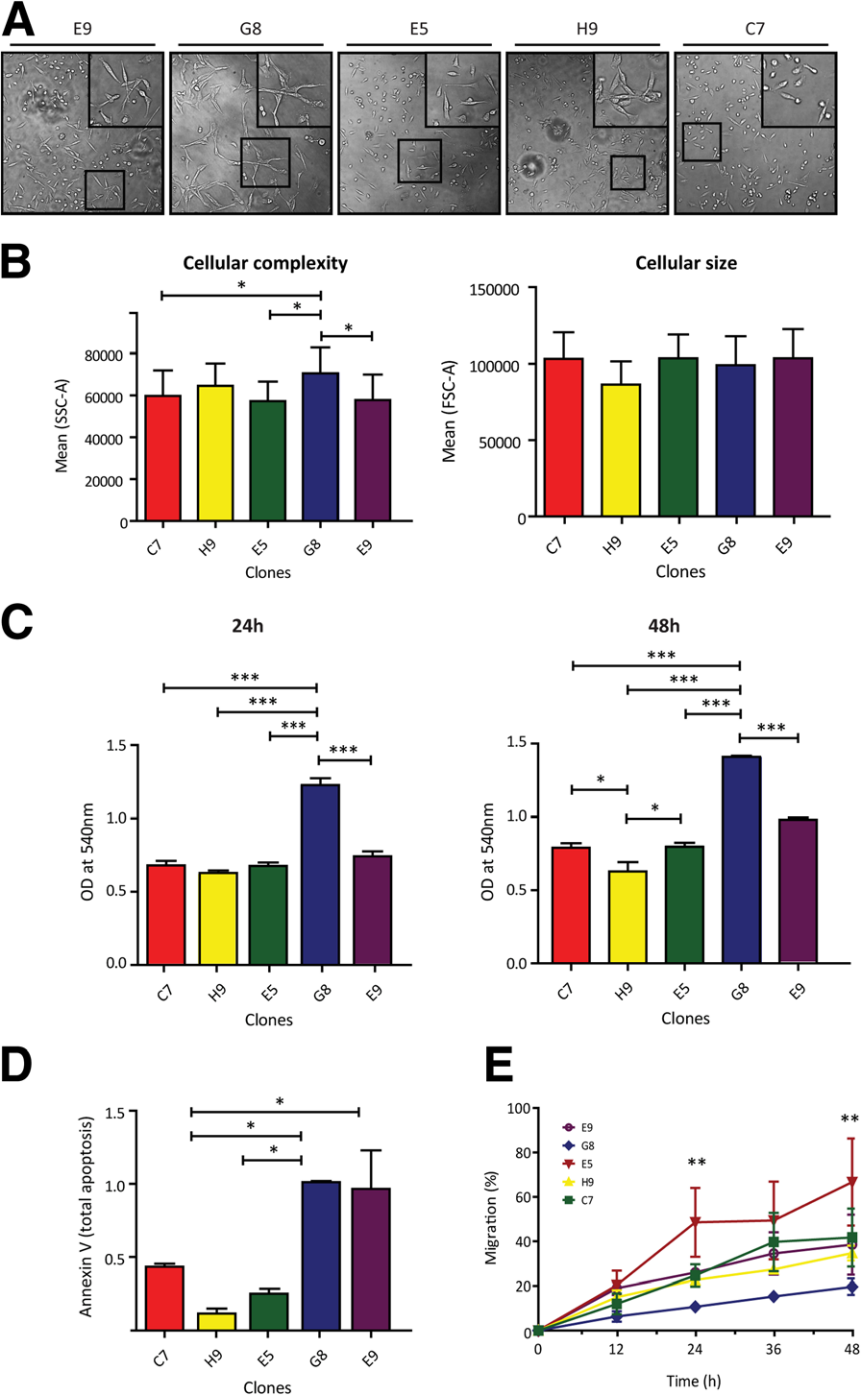
Morphology and behavior features of cellular clones derived from MDA-MB 231 cancer cell line. **a.** Optical microscopy images showing the growth pattern of different MDA-MB 231 derived clones. While C7, E5 and E9 clones presented fibroblast-like phenotype (isolated cell grown pattern), H9 and G8 clones presented epithelial like phenotype (grouped cell pattern) (figure insets). **b.** As can be observed in flow cytometry analysis, the clones present significant differences in cellular complexity and not in cellular size (SSC and FSC respectively) (ANOVA with Tukey post hoc test *p* < 0.0016). **c.** Bar graph showing significant differences in the proliferation rate of the different clones after 24 h by MTT assay (left graph). The differences are increased at 48 h (right graph) (ANOVA with Tukey post hoc test p < 0.0001). **d.** Bar graph describes significant differences of apoptosis response after 30 min of UV exposure (Anexin V/PI test) (ANOVA with Tukey post hoc test *p* < 0.0173). **e.** Migration of different clones was tested by wound-healing assay at 12, 24, 36 and 48 h. As can be observed, clones presented significant differences at 24 and 48 h of migration (ANOVA pos-hoc Tukey test *P* < 0.0021). Asterisks in all panels indicate significant differences (*, *p* < 0.05; **, *p* < 0.01; ***, *p* < 0.0001)

Five clones were selected with detectable differences in their proliferation rate (C7, H9, E5, G8 and E9 clones). After clone expansion, we observed that some of them presented different growth patterns. In addition, some clones displayed fibroblast-like phenotype (C7, E5 and E9) and others epithelial-like phenotype (H9 and G8) (Fig. 3A). Interestingly, each clone preserved phenotype and growth pattern along time (2 months, data not shown). We also studied morphological characteristics by flow cytometry. Significant differences were detected in internal cellular complexity (Side scatter) and no significant differences in cellular size (Forward scatter) by ANOVA test (*P* = 0.0016) and (*P* = 0.645) (Fig. 3B). These observations led us to ask whether the clones could present different behavior. When we studied the proliferation rate after 24 h and 48 h by MTT assay, we found significant differences among all the clones as compared to the initial population at 48 h (ANOVA *p* < 0.001) (Fig. 3C). We also evaluated the apoptosis response to 30 min of UV irradiation using Annexin V/PI assessed by flow cytometry. As in the proliferation assay, we observed significant differences among the cellular clones (*p* = 0.01) (Fig. 3D). Finally, migration capacity of different clones was tested by wound-healing assay at 12, 24, 36 and 48 h. Again, some clones presented significant differences at 24 and 48 h of migration (ANOVA pos-hoc Tukey test *P* < 0.0021) (Fig. 3E).

These results suggest that the initial population of MDA-MB 231 cells is epigenetically heterogeneous and is composed by different clones that show diverse morphology and behavior.

### MDA-MB 231 derived clones are heterogeneous in epigenetic terms

**Fig. 4:**
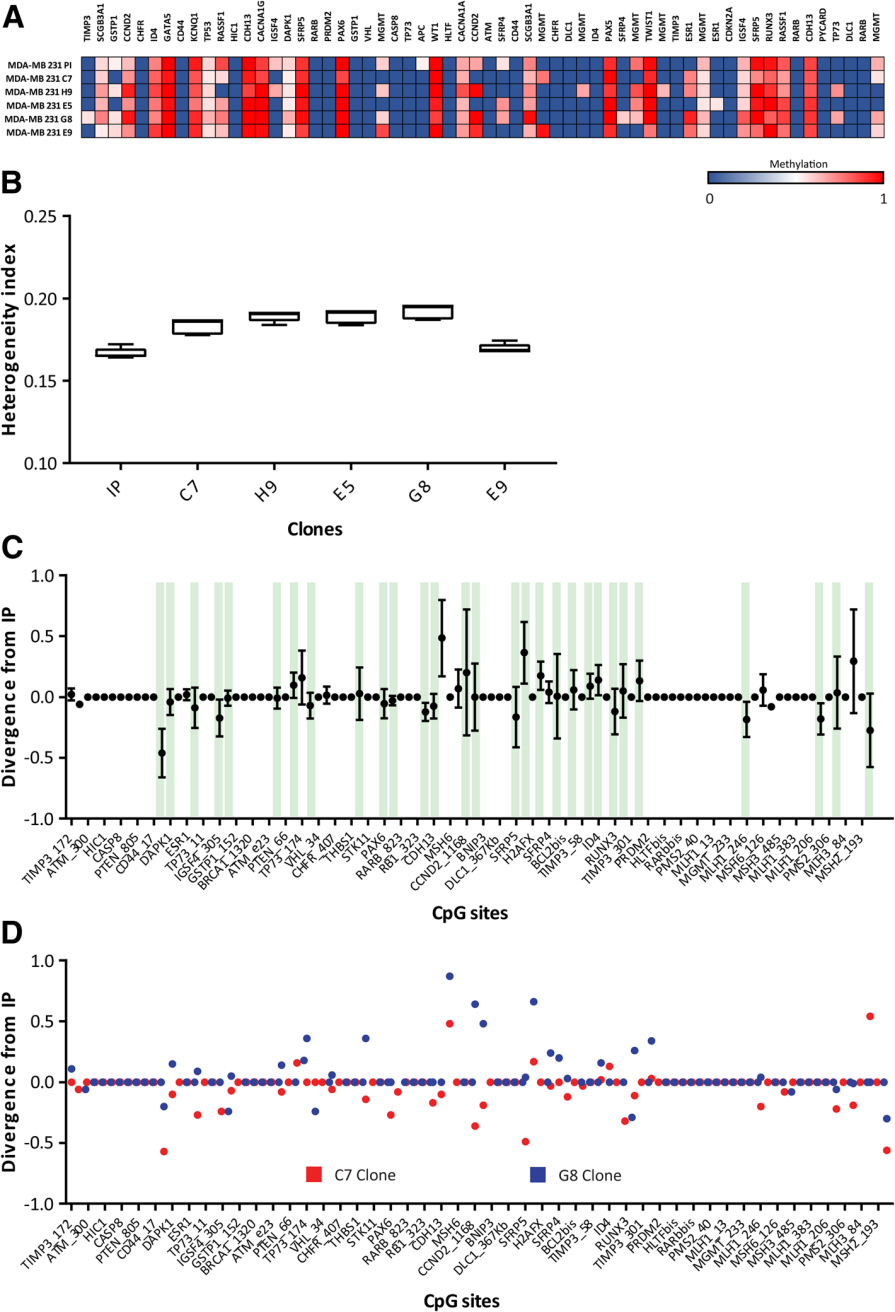
Heterogeneity index of MDA-MB 231 clones inferred from methylation profile obtained by MS-MLPA. **a.** Heat map showing the methylation status of 60 CpG sites (in columns) on MDA-MB 231 initial population and 5 clones (in rows). A color gradient from blue-white-red is used to represent low to high values of methylation (from 0 to 1). **b.** HI calculated for the MDA-MB 231 initial population (IP) and 5 clones represented in box plot graph. **c.** Scatter graph showing the methylation divergence from IP of 60 CpG sites for all the clones. Divergence was defined as the difference of methylation value of the CpG sites between the clones and the initial population (∆ = Clone - IP). Green bars show the CpGs methylated in the IP. **d.** Scatter graph showing the individual divergence of two clones. G8 clone (blue dots) diverged mostly increasing the methylation while C7 clone (red dots) diverged mostly decreasing the methylation of CpG sites

The next question was if the clones were homogeneous in epigenetic terms. To address this question, we studied the methylation profile of the cellular clones by MS-MLPA. We observed different methylation patterns among clones which differed also as compared to initial population of MDA-MB 231 cells. Several CpG sites presented intermediate and different methylation values (between 0 and 1); which meant that these sub-populations derived from MDA-MB 231 were heterogeneous (Fig. 4A). We wondered which were the CpG sites that changed in the clones with respect to the IP. The rationality behind this was the idea that probably not all methylation sites would be able to modify the epigenetic landscape or generate equal advantages. We detected that each CpG site presented different dispersion between the distinct populations. Indeed, we observed that some sites (e.g. SCGB3A1, CCND2, ID4, GATA5, TP53, KCNQ1, CDH13, CACNA1G, SFRP5, PAX6, WT1, CACNA1A, SCGB3A1, PAX5, TWIST1, IGSF4, SFRP5, RUNX3, RASSF1 and CDH13) were methylated in all the clones (in different proportions) and other sites were methylated in some clones (TIMP3, GSTP1, RASSF1, IGSF4, MGMT, CCND2, SFRP4, ESR1 and TP73) and unmethylated in others. Moreover, we detected methylation of some CpG sites that were not methylated in IP (TIMP3, MGMT, SFRP4, ESR1, and TP73). This observation suggests that the clones presented a different epigenetic profile respect to the IP; it is important to remark that not all the methylation sites underwent modifications to recreate heterogeneity in the one-cell derived clones.

We applied the HI calculation to measure the magnitude of the heterogeneity of the clones derived from MDA-MB 231 (Fig. 4B). Initially, we thought that clones would exhibit low heterogeneity. Unexpectedly, and despite the fact that clones had been originated from a single cell, the HI of these populations did not differ from the HI of the IP. Interestingly, they concentrated in a narrow range (0.16–0.19) around the HI of the IP.

From this analysis we could infer that, after 30–60 days (depending on the proliferation rate of each clones), sub-clonal cell populations generated from a single cell of a well-established breast cancer cell line would develop different methylation patterns. Interestingly, according to the HI, the clones presented an epigenetic heterogeneity with a similar magnitude of the IP.

### Clones derived from MDA-MB 231 cells show epigenetic divergence without new selective pressure

As we mentioned above, the clones were obtained from a single cell and were grown under controlled and non-competitive conditions for two months. As mentioned previously, we observed that the clones presented epigenetic heterogeneity despite no apparent new selective pressure was applied. So, we aimed to unravel if the heterogeneity observed in vitro is generated by epigenetic divergence of the clones.

To address this question, we defined epigenetic divergence as the difference between the clones and the IP of the 25 CpG sites methylation values (∆ = Clone - IP) (Fig. 4C). Interestingly we observed a slight tendency to a positive divergence (hypermethylation) since 21 CpGs increased the methylation proportion versus 18 CpGs which diverged to unmethylation. However, if we analyze the divergence at individual level, we observed that each clone had its own divergence tendency. For example, the G8 clone (blue dots) diverged preferably by increasing the methylation while C7 clone (red blots) diverged rather by decreasing the methylation of CpG sites (Fig. 4D).

Another interesting observation was that most DNA methylation changes occurred in CpGs already methylated in the IP (Fig. 4C, green bars), suggesting that clones are the expansion of cell types that are representative of the prevalent population of the IP. An attractive exception was the methylation of ESR1, all clones presented methylation of this gene while it was unmethylated in the IP. All together, these evidences suggest that the methylation is not a random event.

Based on these observations, we conclude that the clone populations generated their own new heterogeneity mostly by altering the methylation state of CpG sites already methylated in the IP.

### HI correlates with clinic-pathological features of breast tumors

**Fig. 5:**
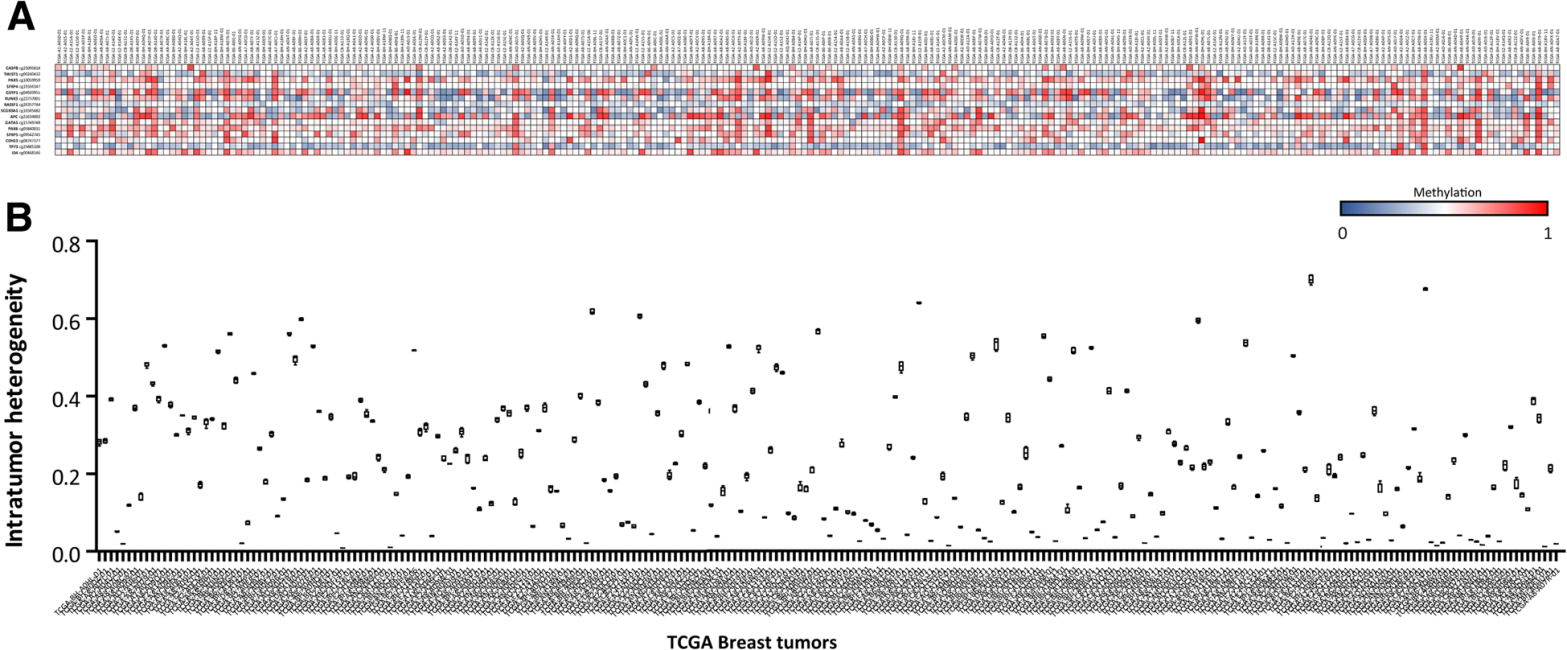
Heterogeneity index of 250 breast cancer tumors inferred from methylation profile obtained from TCGA dataset. **a.** Heat map showing the methylation status of 15 CpG sites (in rows) on 250 breast tumors (in columns). A color gradient from blue-white-red is used to represent low to high values of methylation (from 0 to 1). **b.** HI calculated for the 250 breast tumors represented in box plot graph

We measured intratumor epigenetic heterogeneity in IDC from patients as well as in cellular clones. Since the clones HI were associated with different behavior, we aimed to see whether the HI of tumors could be associated to clinical-pathological features. We decided to test this hypothesis using a public resource such as TCGA which collects the data from several studies, with 250 tumors (Fig. 5A) and complete clinical-pathological information.

We selected 15 CpG sites for the HI calculation in TCGA tumors (included in Table 2). Again, the selection was based on the most frequently methylated CpGs. As in the fresh tumor cohort, we observed a great dispersion in the HI obtained for the TCGA tumors (Fig. 5B). A significant positive correlation was found between HI and “Estrogen Receptor expression” (r = 0.369 *p* < 0.0001) and “HER2 status” (r = 0.2264 *p* = 0.0007).

Another remarkable observation was the positive correlation with “tumor stage” (r = 0.1678 *p* = 0.0121), “number of lymph nodes affected” (r = 0.2048 *p* = 0.007) and “tumor size” (r = 0.1713 *p* = 0.0088).

These interesting results revealed an association between the epigenetic intratumor heterogeneity and relevant prognostic and predictive factors (like tumor size, number of affected axillary nodes and Estrogen receptor and HER2 positivity).

### Different cellular tumor populations share epigenetic heterogeneity index

Until here, by using a mathematical model we inferred intratumor epigenetic heterogeneity from methylation data derived from fresh IDCs, cancer cell lines, cellular clones and TCGA tumors.

When comparing the information obtained from these diverse sources, we observed that the average HI was similar (ANOVA *p* = 0.53), i.e.: fresh tumors = 0.1504, cancer cell lines = 0.1888, cellular clones = 0.1811 and TCGA tumors = 0.1979. Notice that we employed different number and different locations of CpG sites for the HI calculation on the distinct populations, making this convergence more surprising. In addition, surgical tumor margins presented a significantly lower HI (0.0049) (*p* < 0.001). So, it is reasonable to propose that tumor cell populations share a similar HI which differs from healthy cellular populations.

**Fig. 6:**
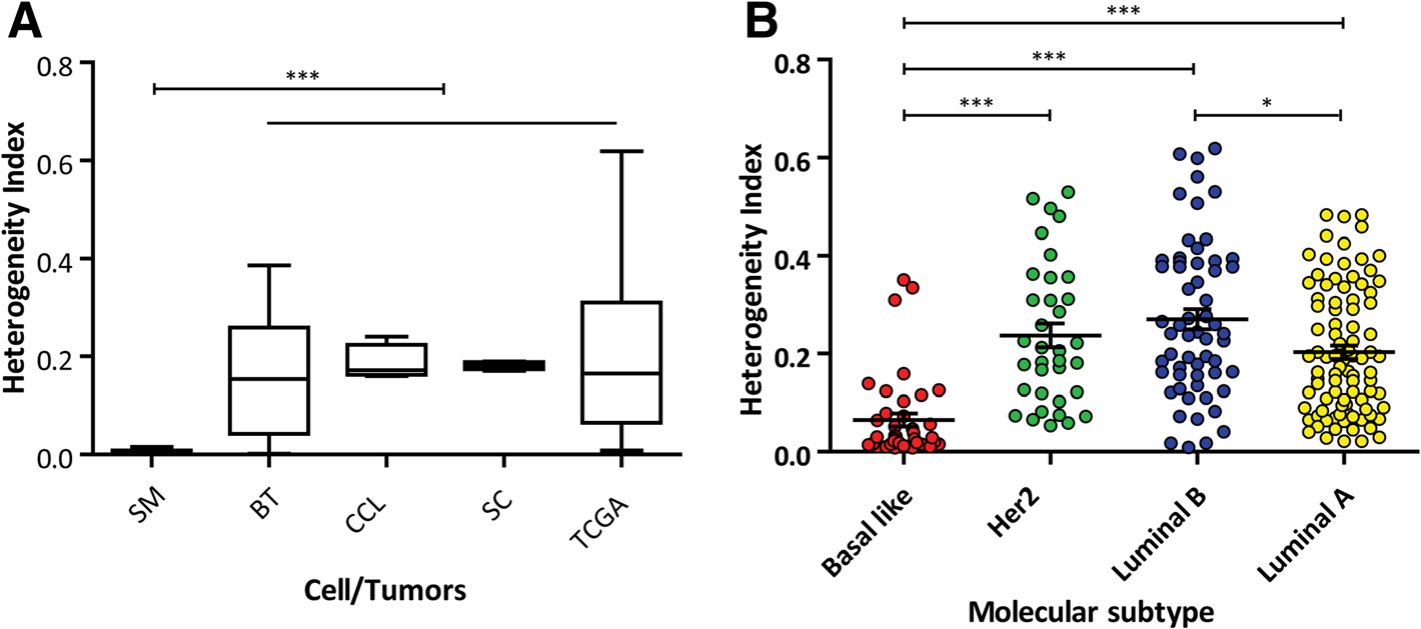
Heterogeneity index average of cancer cell populations and normal cells obtained by MS-MLPA. **a.** Average HI for the different populations: breast tumor surgical margins (SM), breast tumors (BT), cancer cell lines (CCL), MDA-MB 231 clones (SC) and breast tumor TCGA dataset (TCGA). The HIs are statistically different as assed by ANOVA with Tukey post hoc test *p* < 0.0001. **b.** Grouped column scatter graph showing the mean and SEM of different subtypes of the TCGA tumors (ANOVA with Tukey post hoc test *p* < 0.0001)

Interestingly, we observed that the distinct molecular tumor subtypes (Basal like, Her2, Luminal A and Luminal B) presented different heterogeneity levels (ANOVA analysis *P* < 0.0021) (Fig. 6B). By deepening in these observations, it is worth to notice that fresh tumors and TCGA tumors present a broad deviation from the mean. In line with this, when we ranked the HI values in “low” (from 0 to 0.17), “medium” (from 0.18–0.22) and “high” (above 0.22) we found that tumors were not distributed at random among these ranges. Most of the tumors (90%) were distributed in the “low” or “high” rank, whereas only 10% was included in the “medium” rank. In addition, a significant association was detected between the HI ranks and the tumor subtype PAM50 classification, i.e.: 38/ 42 (90%) of basal like tumors presented “low” HI, while Luminal A and HER2 tumors were mostly equally distributed among “low” and “high” ranks; beside, more than the half (58%) of Luminal B tumors were included in the “high” range of HI (Table 3).

**Table 3:** Molecular subtypes of breast TCGA tumors

		Tumor subtype (PAM50)				
		Basal like	HER2	Luminal A	Luminal B	Total
Grouped HI	Low (0–0.17)	38	13	45	18	114
	Medium (0.18–0.22)	0	7	11	7	25
	High (0.23–0.7)	4	15	35	34	88
		42	35	91	59	227

We consider these observations very attractive since it suggests that, depending on the tumor type, the HI is deviated from the mean, increasing it in Luminal A, B, Her2 or decreasing it for basal like sub types.

## Discussion

A tumor piece examined by a pathologist in a stained tissue or on a slide glass, is the final picture of an evolution process that started with a normal cell which acquired consecutive genetic and epigenetic alterations over time and as in all evolution processes, the outcome constitutes a heterogeneous population [27] [28] [3]. The resulting intratumor heterogeneity is one of the principal factors involved in resistance to standard oncological treatments and in tumor progression [4]. Several studies in solid tumors have addressed the intratumor heterogeneity as inferred from genetic mutations [29] [30], gene expression [31] [20] or protein modifications [32] [33]. Epigenomic alterations, which contrast with genomic alterations because of their dynamism and reversibility, evolve during a tumorigenic process in parallel to the acquisition of driving and passenger mutations [34]. Therefore, they are relevant information for the study of spatial and temporal intratumor heterogeneity.

Different research works have described that cancer cells with specific mutations, present prominent sub-clonal diversification and that most of the genetic alterations are found in a fraction of the tumor [35] [36]. On the other hand, some authors have shown epigenetic heterogeneity by analyses of CpG methylation in different fractions of the same tumor in prostate and breast cancer [34] [37]. In this work we aimed to establish the intratumor heterogeneity magnitude as inferred from DNA methylation data.

We developed a heterogeneity index employing multiple markers (CpG sites) distributed in different regions of the human genome to quantify for the first time the epigenetic intratumor heterogeneity in breast cancer. For this, we established a mathematical model which conjugates the methylation information of multiple CpG sites located in promoter regions of TSG to generate a numerical index of heterogeneity of distinct cellular populations derived from different clinical and experimental models of breast cancer. It is important to stress that the CpG sites included in the index calculation are normally unmethylated in healthy cells, but frequently methylated in cancer cells. Therefore, alleles derived from the non-tumor cells only have a dilution effect in the index calculation. The HI can be also influenced by the copy number of the tumor cells because it pools together intra and inter cellular methylation heterogeneity. Considering this, tumors with multiple copy number alterations were excluded from this analysis. We also tested that CpG sites involved in the heterogeneity index calculation did not have significant copy number amplification in cancer cell lines nor in tumors. This is consistent with the fact that it is unlikely that TSG regions would be amplified in tumor cells [38] [39].

The starting point of this study was the observation of intermediate methylation values for multiple CpG sites in fresh IDC fractions derived from patients with breast cancer analyzed by MS-MLPA [16]. The same phenomenon can be observed in beta values of TCGA datasets, where DNA methylation profiles were measured experimentally using the Illumina Infinium HumanMethylation450 platform. Although the intermediate values of TSG methylation indicate ITH by themselves, the quantification of the heterogeneity allowed us to determine that not all tumors present the same level of heterogeneity. According to our hypothesis, using different sources of epigenetic information and different number of CpG sites, the HI obtained for each tumor was different. In fact, HI values presented high dispersion in both fresh and TCGA tumor cohorts. Therefore, if each tumor presents a specific heterogeneity value, this means that HI could be considerate as an individual tumor feature.

When we analyzed the HI in two different tumor cohorts (fresh tumors and TCGA tumors), we were surprised by the observation that they presented a similar HI mean (as well as in other tumor cell populations, discussed below), in contrast with the non-tumor population derived from de surgical margin which presented a lower HI mean. We previously mentioned that only 10% of the TCGA tumors presented HI values around the mean (40% of tumors exhibit lower and 50% higher HI than the mean value). According to Nowell’s model, these observations invite to propose an evolution mechanism involved in the generation of the ITH. Although more evidence is necessary to clearly explain this phenomenon, we propose to analyze the tumor cells as an ecological population where the local microenvironment shapes the epigenetic landscape leading the cell population to establish heterogeneity levels above or below a mean value. The same external conditions would create a selective pressure responsible for the establishment of different levels of HI. Higher HI values describe tumors with greater diversity among their cells, indicating probably the existence of a divergent selection pressure on the population. Divergent selection pressure is known to depress the adaptability of the individuals (or cells in this case) which represent the mean values, by favoring sub-groups with values distant from the mean. This kind of selective pressure disrupts the population maintaining high levels of divergence. On the contrary, lower HI values can be considered as indicative of populations tending to a homogenous value among the cells, where variability is not favored. This could be the case of a directional selective pressure, by which the population is shifted to a specific value and cells which carry this feature are positively selected. In this case, the tumor population is adapted through the acquisition of a specific feature, and therefore the HI is decreased among the cells.

It has been described that although cancer cell line cultures do not reproduce cellular tumor architecture and intratumor heterogeneity, this experimental model could provide information on the clones functional features [40]. We detected ITH in different cancer cell lines and observed that the mean value of HI between these cell lines was similar to the mean HIs observed in both tumor cohorts. However, the dispersion of the HIs in cancer cell line populations was lower than the dispersion observed in tumors. This evidence is consistent with the fact that no apparent selective pressure is applied over cell culture populations, or that directional and divergence selective pressures are equated; in contrast to the tumors, where tumor cells are exposed to a strong environment constraint. Contrary to our speculations, clone populations derived from one of these cell lines, showed similar levels of heterogeneity than the initial population. This means that the generation of epigenetic heterogeneity in populations derived from a unique cell is possible after 1–2 month of proliferation. We determined divergence in epigenetic terms and different behavior of clones in the absence of specific selective pressure. Similar evidences were described in phenotypic and functional heterogeneity for different clones derived from single cell of MDA-MB 231 and MCF-7 cell lines at early stages of clonal development [41]. The HI mean value of the clone populations was similar to the other cancer populations (cell lines and tumors), however the HIs presented a lower dispersion even than cancer cell lines.

Taking into consideration the observation in cell lines and in breast tumors, we think that there is a mixed phenomenon, where the external factors affect the methylation landscape available to the cells and contribute to select and establish an epigenetic heterogeneity. Since in vitro experiments with cancer cell lines (5 cell lines and 5 clones), where no specific selective pressure was applied, and similar HIs were observed, we propose that in breast tumors the microenvironment acts over a basal heterogeneity characteristic of each tumor cell population.

It has been reported that tumors harboring high levels of genetic ITH were associated with more aggressive disease progression [42]. In this work we determined that early-stage tumors presented lower ITH levels in TCGA breast cancer tumors. However, the group of breast carcinomas with higher HI were enriched with luminal A, luminal B and HER2 phenotypes which have a better prognosis than those with basal like phenotype [43]. In contrast, around 90% of basal like breast tumors was in the group with lower HI. Our hypothesis is that basal like tumors presented a lower variability, probably as a consequence of a directional selection process. It is important to mention that similar tendencies of association were observed between HI levels and tumor stage or ER and HER2 expression when we analyzed the fresh tumor cohort (data not shown).

Different measurement methods have been recently developed to express the observed intratumor heterogeneity as a numerical value [44] [45] [46]. The challenge is to obtain a useful clinical biomarker which could predict the risk of progression or response to treatment. The HI developed in this work contributes with this purpose and can be calculated using methylation profiles obtained with different methodological approaches. We consider that a deep exploration of this tumor feature using for example mathematical tools, is crucial both to unravel the mechanisms implicated in the spatial and temporal intratumor heterogeneity development and to improve the study of clinical tumor progression and treatment.

## Conclusions

We developed an index to quantify for first time epigenetic heterogeneity in solid tumors inferred from tumor suppressor genes methylation patterns in different tumor cell populations (i.e fresh breast carcinomas, cancer cell lines and breast TCGA tumors). By applying the develop HI calculation, our analyses allow to conclude that all studied tumors or cultivated cells present heterogeneity, suggesting that epigenetic homogeneity is avoided during tumorigenesis. And even though we could establish that each tumor presents unique HI, our work shows also that some tumors share similar levels of HI in association with their subtype classification. We therefore conclude that tumor subtype’s behavior could be described in terms of epigenetic heterogeneity, which could serve as a new contribution to understand the different prognosis of these groups.

## Abbreviations

HI: Heterogeneity index
IDC: Invasive ductal carcinoma
TCGA: The Cancer Genome Atlas
TSG: Tumor suppressor genes
